# Quality Control Technology for Abrasive Flow Precision Machining of a High-Performance Impeller

**DOI:** 10.3390/mi16121370

**Published:** 2025-11-30

**Authors:** Junye Li, Songyuan Li, Pingping Wei, Changqing Wang, Yanming Li, Ke Liu, Chunlin Liu, Yu Chen, Guiling Wu, Xiao Li, Baicheng Liu, Jiyong Qu, Haihong Wu, Jun Zhang, Ziqiang Zhang

**Affiliations:** 1 Ministry of Education Key Laboratory for Cross-Scale Micro and Nano Manufacturing Changchun University of Science and Technology, Changchun 130022, China; ljy@cust.edu.cn (J.L.); songyuan_li@163.com (S.L.);; 2 Changchun Jianzhen Precision Machinery Manufacturing Company Limited, Changchun 130212, China; wpp980890@163.com; 3 AECC Harbin Dongan Engine Company Limited, Haerbin 150066, China; 4 China North Vehicle Research Institute, Beijing 100072, China; 5 Jianglu Machinery Electronics Group Company Limited, Xiangtan 411100, China; 6 AECC Changchun Control Technology Company Limited, Changchun 130052, China

**Keywords:** solid–liquid two-phase abrasive flows, surface quality, numerical simulation

## Abstract

The surface quality of high-performance impellers, which feature complex, free-form surfaces and narrow flow channels, is critically important for their performance and efficiency. However, achieving uniform precision polishing on these intricate geometries remains a significant manufacturing challenge, as traditional methods are often inefficient, inaccessible, or cause surface damage. To address this, this study investigates the application of solid–liquid two-phase abrasive flow machining (AFM) as a high-precision finishing solution. Through numerical simulation, we analyzed the polishing effects under two flow channel structures and various machining parameters. The results demonstrate that a gradual flow channel structure significantly enhances processing uniformity and intensity compared to a direct flow channel. Furthermore, increasing the inlet pressure and abrasive viscosity was found to substantially improve both the strength and uniformity of the machining effect across the impeller surface. Experimental validation via an orthogonal test design confirmed that inlet pressure is the most influential factor on the polishing effect, followed by abrasive grain size and the number of processes. The optimized process parameters (6 MPa inlet pressure, 10 process cycles, and 40 µm abrasive grain size) successfully reduced the average surface roughness (Ra) of the high-performance impeller from 0.766 µm to 0.047 µm, representing an improvement of nearly 94%. This study provides a scientifically grounded set of optimal parameters for achieving uniform, high-quality surface finishing of complex impellers using AFM technology.

## 1. Introduction

As the key component of power machinery, the impeller has the advantages of excellent working performance and long service life. It is extensively used in aerospace, military, automotive, and other industries. Kang and Kim, et al. aimed to analyze the effects of dynamic motion and structural response of semi-submersible floating offshore wind turbine structures (FOWTs) in waves generated in hurricane environments [1]. However, the surface quality and precision of high-performance impellers can directly affect the overall power performance and mechanical efficiency of machinery [2]. Similarly, as an important transmission component of the shielded motor pump, the impeller works in a high-pressure and high-speed rotation state. The quality and performance of the impeller surface determine the overall operating condition and service life of the shielded motor [3]. The impeller is the core component of an aero motor, and its blade is mostly free surface. Polishing material uniformly to achieve impeller high-precision surface processing helps in improving impeller workpiece product performance and service life, and improves aero-engine efficiency [4]. However, high-performance impellers are difficult to machine precisely due to the presence of complex wheel surfaces, which are usually free-form and contain narrow flow channels, severe blade distortion, comparatively long blades, low stiffness and other defects. Modern processes for workpiece surface polishing and finishing technology mainly include CNC mechanical polishing [5], abrasive belt polishing [6], chemical electrolytic polishing [7], micro- and nanomachining [8,9] and laser-assisted machining [10]. However, CNC mechanical polishing efficiency is low and polishing quality is unstable; abrasive belt polishing cannot meet the requirements of complex internal surface processing; chemical electrolytic polishing is unsteady and pollutes the environment; and micro- and nanomachining and laser-assisted machining are expensive and difficult to execute [11]. As a result, in order to solve the manufacturing and processing issues of high-performance impellers, a highly efficient, high-precision and low-damage machining technique must be investigated.

Abrasive flow machining (AFM) is the repeated flow of viscoelastic abrasive through the surface of a workpiece using a power source to complete the finishing work, which can achieve the requirements for removing excess material from the surface of the workpiece, surface refinement and other aspects, effectively improving the surface quality of complex surfaces [12,13,14]. Zhu and Sun, et al. found that ATP abrasives can be used in abrasive air jets (AAJ) to remove coatings from GFRP surfaces. The results of this study showed that ATP abrasive can not only be used in AAJ (about 15 cycles of use) to remove the coating by delamination, but also can avoid damage to the GFRP substrate surface [15]. Gao and Guo, et al. used rotary tap tools as the research object and adopted an orthogonal experimental design method to investigate the effects of three factors, namely, tool rotation speed, polishing time and abrasive mass fraction, on the surface finish in the process of abrasive flow polishing. A mathematical model was established with the surface finish as the optimization target. The results showed that the surface roughness could be reduced from 0.73 μm to 0.26 μm, and the radius of edge rounding could be controlled within 5 μm as required by the enterprise, which avoids the phenomenon of “overtoning” of the tool edge [16]. Toykoc and Basu, et al. present a multi-physics simulation model of the AFM process that predicts the interactions between heat generation, heat transfer, media temperature, and media flow. Predictions of local pressure and temperature within a flow channel showed very good agreement with measurements derived from AFM experiments [17]. Nagalingam and Toh, et al. are developing a novel multi-jet hydrodynamic cavitation abrasive finishing (MJ-HCAF) method for polishing complex L-PBF components to improve surface quality [18]. Zhang and Ying, et al. used the abrasive flow etching method to process the surface of titanium alloy flakes and investigated the effect of various roughness values on the cell adhesion properties of the flake surface. The experimental results showed that the surface uniformity of the titanium alloy flakes was good after the abrasive flow processing treatment; the adhesion rate of the flakes was the highest when the surface roughness ranged from 0.08 μm to 0.10 μm, and the adhesion effect was the best [19]. Zhu and Wu, et al. compared particle ploughing and sliding processes, finding that particle micro-cutting and indentation processes have an obvious effect on the surface residual stress; furthermore, the particle micro-cutting process that can create an impacted surface with high material deformation and ductility is more beneficial for improving the compressive residual stress than the particle indentation process [20]. Zhang and Yuan, et al. proposed a high-speed abrasive particle flow compound polishing method based on the dielectrophoretic effect, and conducted high-speed abrasive particle flow observation experiments. The experimental results show that the method can effectively improve the adsorption quality of SiO_2_ abrasive particles, with an abrasive particle removal efficiency of up to 98% [21]. Poudel and Lee, et al. present an innovative approach to enhance the MAF performance by adding nano-scale solid lubricant into the brush. The new MAF brush is also expected to improve the efficiencies of the overall MAF process in terms of energy consumption and material utilization [22]. Ji and Qi, et al. performed abrasive flow machining to smooth up the surface of a titanium alloy artificial knee joint. The workpiece’s surface roughness was reduced from approximately 394 nm to 171 nm, and the homogeneity was improved [23]. Wang and Yang, et al. present a new technique for dressing using abrasive water jets. The results show that the dressing effect of the abrasive water jet is good. The dressing quality first increases and then decreases with increasing jet pressure, nozzle target distance and injection angle, and increases with the increase in abrasive concentration [24]. Yahya and Reza, et al. proposed the ultrasound-assisted rotational magnetorheological abrasive flow finishing (UA-RMRAFF) process, which involves applying ultrasonic vibrations to the workpiece in a direction perpendicular to the magnetorheological polishing (MRP) fluid flow direction. Their experimental findings illustrate that the UA-RMRAFF process provides a uniform and fine surface finish without surface defects such as a mirror up to the range of 25.5 nm [25]. Basha and Venkaiah, et al. independently developed a long-lasting and corrosion-resistant grinding media and used it in the finishing process. The resulting surfaces reduced Ra by up to 90% in the longitudinal direction and 75% in the transverse direction [26]. Therefore, it is especially necessary to investigate the effectiveness and rationality of abrasive flow polishing of high-performance impellers, as well as the influence of processing parameters on the surface quality of high-performance impellers.

This study investigates the feasibility of solid–liquid two-phase abrasive flow precision machining technology in the application of high-performance impeller complex curved component polishing. By comparing the influence on the surface quality of the impeller under two different process conditions, namely, the direct flow channel and the gradual flow channel, the influence on the surface quality of the impeller is investigated. On the basis of determining a perfect flow path, the influence of important elements, including inlet pressure, inlet speed, and abrasive viscosity, on the surface finish of high-performance impellers is carefully explored. The experimental results illustrate that, when compared to traditional processing, abrasive flow polishing significantly improves the surface quality of the high-performance impeller, providing a scientific foundation for the engineering application of this precision processing method.

### Innovation Points of This Study

In response to the above limitations, the core innovations of this article are as follows:

(1) Gradient channel structure innovation: For the first time, a gradient contraction type channel is designed to enhance the local abrasive collision strength through spatial gradient constraints, solving the weak problem of traditional straight channel blade root processing.
(2) Quantitative innovation of coupling mechanism: We break through the bottleneck of single-parameter qualitative analysis and reveal the synergistic effect law of pressure, velocity, and viscosity, establishing quantifiable parameter regulation criteria.
(3) Innovation of quality control system: We integrate orthogonal experiments and nonlinear regression to construct a closed-loop system of “parameter optimization quality prediction effect verification”, replacing the experience-dependent processing mode.

## 2. Equations of Solid–Liquid Two-Phase Flow

The fluid in a solid–liquid two-phase abrasive flow is recognized as an incompressible continuous viscous fluid, which means that the volume or density of the fluid remains constant throughout the flow. The physical conservation laws that should be followed during abrasive flow include the continuity equation, the momentum equation and the energy equation.

The continuity equation expresses the rule of conservation of mass in fluid motion. Then the expression for the conservation of mass of the system is as follows:

(1) $$\frac{dm}{dt}=\frac{d}{dt}{∭}_{V\left(t\right)}\rho dV=0$$

where the volume of fluid at time t = t is V(t), the mass is m, and the density is ρ.

Applying the Reynolds transport equation we obtain the following:

(2) $$\frac{d}{dt}{∭}_{V\left(t\right)}\;\rho dV={∭}_{V\left(t\right)}\left[\frac{\partial \rho }{\partial t}+\nabla (\rho \vec{v)}\right]dV=0$$

The conservation of momentum equation is a manifestation of the principle of conservation of momentum in fluid motion. We assume that the force per unit volume of matter is $\rho \vec{f}$. On the boundary S(t), the force per unit area of matter is $\vec{p}$. In the region V(t), the momentum of matter is ${∭}_{V(t)}\rho \vec{v}dV$. The momentum equation can be written as follows:

(3) $$\frac{d}{dt}{∭}_{V\left(t\right)}\rho \vec{v}dV={∭}_{V\left(t\right)}\rho \vec{f}dV+\iint_{S\left(t\right)}\vec{p}dS$$

Applying Reynolds’ transport theorem, the conservation of momentum equation is obtained as follows:

(4) $${∭}_{V\left(t\right)}\rho \frac{d\vec{v}}{dt}dV={∭}_{V\left(t\right)}\rho \vec{f}dV+\iint_{S\left(t\right)}\vec{p}dS$$

The conservation of energy equation is the expression of the principle of conservation of energy in fluid motion. Assuming that the only external forces are the volume and area forces, the rate of change of kinetic and internal energy of the substance in the volume V(t) is equal to the work done by the volume and area forces per unit time plus the heat given to the substance by the outside world per unit time. The law of conservation of energy can be expressed as follows:

(5) $$\frac{d}{dt}{∭}_{V\left(t\right)}\rho \left(e+\frac{1}{2}{\vec{v}}^{2}\right)dV=\sum W+\sum Q$$

where e represents the internal energy per unit mass of fluid, and ρe is the internal energy per unit volume of fluid; $\frac{1}{2}{\vec{v}}^{2}$ represents the kinetic energy per unit volume, and the total energy contained in a unit volume of fluid $U=\rho e+\frac{1}{2}\rho {\vec{v}}^{2}$; ∑W is the sum of the work done on the system by internal and external forces per unit time, and ∑Q is the total heat transferred into the system per unit time.

Substituting ∑W and ∑Q into the equation, the law of conservation of energy can be expressed as follows:

(6) $$\frac{d}{dt}{∭}_{V\left(t\right)}\rho \left(e+\frac{1}{2}{\vec{v}}^{2}\right)dV={∭}_{V\left(t\right)}\rho \vec{fv}dV-\iint_{S\left(t\right)}\vec{pv}dS+{∭}_{V\left(t\right)}\rho qdV-\iint_{S\left(t\right)}\vec{f_{F}n}dS$$

where q is the heat distribution function of the heat transfer into the unit mass per unit time; ${∭}_{V\left(t\right)}\rho qdV$ is the total heat transfer into the radiation per unit time; $\vec{f_{F}}$ is the heat flow vector; and $-\iint_{S\left(t\right)}\vec{f_{F}n}dS$ indicates the heat transfer through the surface per unit time due to heat conduction.

## 3. Numerical Simulation Model Construction

### 3.1. High-Performance Impeller Modelling and Meshing

**Figure 1:**
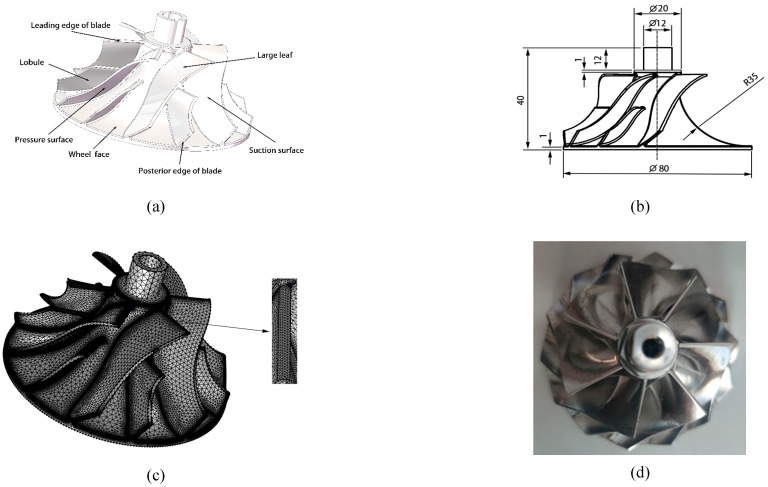
Schematic diagram of a high-performance impeller: (**a**) 3D schematic, (**b**) impeller size, (**c**) impeller meshing, and (**d**) physical view of the impeller workpiece.

High-performance impellers, as key components of power mechanical drives, have important applications in various engineering fields. The 94~03 Ford Powerstroke 7.3 L GTP38 turbocharger impeller (Garrett, Rolle, Switzerland) was selected in this study. The workpiece material is aluminum alloy 2024, which complies with the national standard 06Cr19Ni10, with density $\rho =2.78\;g/{cm}^{3}$, HBW ≤ 145, $\sigma_{b}\geq 425\;Mpa$, and $\sigma_{s}\geq 275\;Mpa$. The high-performance impeller structure consists of three parts (the leaf disc, the large blade, and the small blade), with the large and small blades being equally distributed. The workpiece has a height of 40 mm, an outer diameter of 80 mm, an inner diameter of 12 mm and a bending radius of 35 mm at the hub face. Figure 1 depicts the geometric model diagram, and the impeller faces are named in the diagram in terms of the next step of analysis. Figure 1c depicts the mesh diagram, which has a unit count of 585,896 and an average unit mass of 0.6618. The physical view of the high-performance impeller workpiece is shown in Figure 1d; the units are millimeters.

### 3.2. Flow Chart for the High-Performance Impeller Simulation

**Figure 2:**
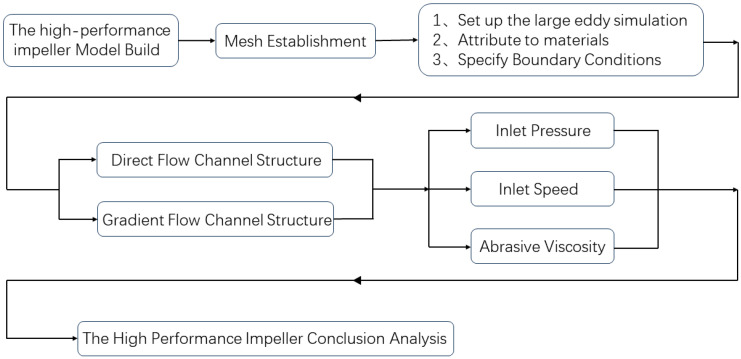
Flow chart for the high-performance impeller simulation.

The three phases of numerical simulation are geometric model pre-processing, solution set-up and result post-processing. The solver set-up process in Fluent software (2022 version) includes factors such as fluid model, material selection, and motion boundary conditions, among others. Only by setting suitable settings in the solution can the flow field changes be adequately depicted, as illustrated in Figure 2.

### 3.3. Analysis of Abrasive Flow Cutting Theory

**Figure 3:**
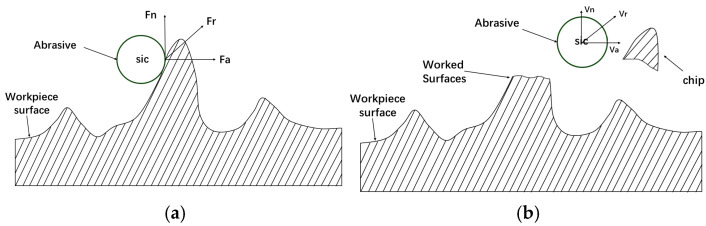
Schematic diagram of the principle of abrasive micro-cutting: (**a**) before cutting, and (**b**) after cutting.

Figure 3 depicts the abrasive flow machining principle. Under the action of external force, the abrasive grains remove the surface material of the workpiece, reducing bumps on the surface of the workpiece to make the surface smooth, and improving the surface quality of the workpiece.

While Fa represents axial force, Fn represents radial force, and Fr represents resultant force. During the abrasive flow machining process, the abrasive grains act as external forces and are pressed into the workpiece surface, resulting in the size and shape of the crater matching the size and shape of the abrasive grains, as well as the magnitude of the external force, and so on. When the abrasive grain acts as an external force on the workpiece surface, it creates a cutting action on the workpiece surface, generates grooves in the workpiece surface, and removes the workpiece surface material, achieving the finishing goal.

### 3.4. Classification of Different Runner Structures

**Figure 4:**
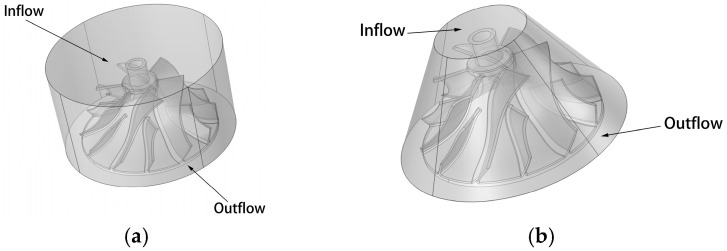
Grid diagram of the flow channel structure: (**a**) direct flow channel structure, and (**b**) gradient flow channel structure.

In the abrasive flow machining process, the workpiece of the high-performance impeller needs to be positioned in order to guide the abrasive to the machined part. A reasonable abrasive flow processing channel can effectively guide the abrasive to reach the processed components and play the role of abrasive finishing, so it is essential to select a suitable channel structure before the abrasive flow processing. The geometric model must be wrapped to produce a closed flow channel based on the abrasive flow characteristics and the geometry of the high-performance monolithic impeller in order to identify a suitable flow path for polishing high-performance impellers. In order to visualize the internal geometry of the flow channel of the object of study, a Boolean operation is used in the numerical analysis process to generate the computational domain to extract the flow channel in three dimensions. The impeller’s upper end is the abrasive inflow port, while its lower end is the abrasive flow outlet, as shown in Figure 4.

### 3.5. Mesh Analysis of Different Flow Channels

**Figure 5:**
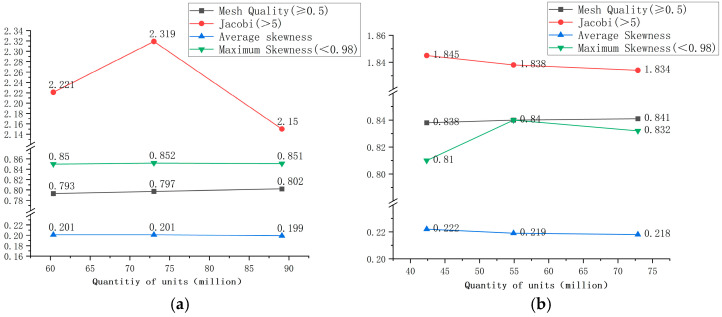
Mesh parameter diagrams for different channels: (**a**) direct flow channel mesh, and (**b**) gradient flow mesh.

The quality of the meshing quality results will determine if the fluid calculations perform well and whether the numerical results are accurate. The quality-independent detection indexes for meshing include three parameters: mesh quality, Jacobian, and skewness. Among these, the mesh quality should be no less than 0.5, the higher the better; Jacobi values should be kept within a range of 5; and the maximum skewness should not be greater than 0.95, the lower the better. The direct and gradient channels are meshed using a tetrahedral unstructured mesh, and their structural parameters are presented in Figure 5.

Observing the relevant mesh parameters under different flow channels in Figure 5, it can be concluded that the mesh quality is generally high in a particular unit number range, which may meet the requirements of numerical analysis and calculation. The mesh mass, skewness, and Jacobi values do not fluctuate dramatically as the number of meshes increased, but only fluctuated slightly within a relatively stable range, passing the requirements for mesh-independence testing. In order to reduce the calculation time and work while maintaining a reasonable level of calculation accuracy, the quantity of mesh units for the direct flow channel and the gradient flow channel are selected as 603,720,000 and 423,640,000, respectively.

### 3.6. Selection of High-Performance Impeller Boundary Conditions

Combined with the actual abrasive flow process, numerical simulation is carried out using FLUENT 17.2; the 3D double precision unsteady pressure-based solver is selected. The time type is transient, and the gravitational influence is considered during the simulation, with the gravitational direction chosen to be the same as the fluid inlet direction, with a value of 9.81 m/s^2^; the turbulence model is the LES model; and the WMLES model is chosen as the subgrid model. The mixture model is selected based on the two-phase abrasive flow processing characteristics. The solid phase is SiC particles with an average particle size of 80 μm and a density of 3170 g/cm^3^, while the liquid phase consists of aviation kerosene with an average density of 780 g/cm^3^. The pressure inlet and pressure outlet are the kinematic boundary conditions, with the pressure output set to standard atmosphere and the turbulence intensity set to 5%. The wall surface adopts no-slip boundary conditions, the momentum adopts the boundary center difference format, and the interaction between abrasive particles in the abrasive particle flow is ignored. The flow field characteristics in straight and gradient flow channels are first simulated and analyzed in order to choose a reasonable flow channel structure. Then the effects of various influencing factors (pressure, velocity, abrasive viscosity, etc.) on the processing effect of the abrasive flow are investigated based on the flow channel structure.

### 3.7. Grid Division of Channel Structure

The quality independence detection indicators for grid partitioning mainly include the following three parameters: grid quality, Jacobian, and skewness. Among these, the grid quality should not be less than 0.5, and the larger the better; Jacobi is generally controlled within 5; and the maximum skewness cannot exceed 0.95, the smaller the better. A tetrahedral unstructured grid is used to partition the DC channel and gradient channel, and the channel structure parameters are shown in Table 1.

Observing the relevant grid parameters of different channels in Table 1, it can be concluded that the grid quality within the number of units is very good and meets the requirements of the numerical analysis and calculation. Moreover, the grid quality, skewness, and Jacobian values do not change significantly with the increase in grid number, only showing small fluctuations within a relatively stable range, meeting the requirements of grid independence detection. Therefore, the number of elements for grid division of the DC channel and the gradient channel were selected as 603.72 million and 423.64 million, respectively.

## 4. High-Performance Impeller Numerical Simulation

### 4.1. Numerical Analysis of the High-Performance Impeller Under Different Channel Structures

According to previous abrasive flow processing experience, a pressure inlet of 6 MPa, an abrasive volume percentage of 40%, and an abrasive particle size of 80 m were selected for numerical simulation analysis to determine the best flow channel model.

#### 4.1.1. Analysis of Impeller Surface Dynamic Pressure Under Different Flow Path Structures

Dynamic pressure, represented as the kinetic energy per unit volume of fluid particles, is an important factor in ensuring the micro-cutting effect of abrasive particles. Dynamic pressure distribution regularity can more clearly and objectively analyze the degree of good or bad abrasive processing on a high-performance impeller surface; therefore, it is very important to analyze the dynamic pressure distribution during the fluid movement in order to determine the effect of abrasive flow processing. The large and small blades on the impeller structure were extracted for inspection, and the surfaces on the left and right sides of the blades were labeled as suction and pressure surfaces according to the impeller’s working condition.

**Figure 6:**
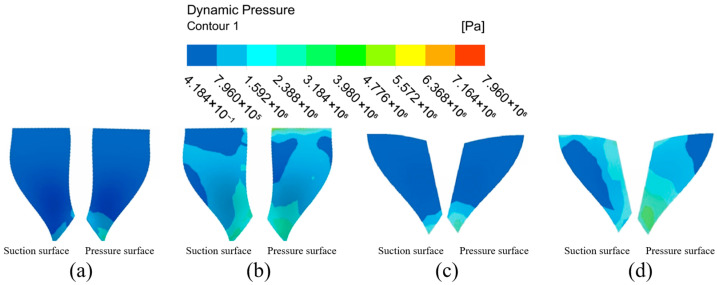
Cloud of dynamic surface pressure distribution with different runner configurations. (**a**) Large blades under direct flow channel structure. (**b**) Large blades under gradient flow channel structure. (**c**) Small blades under direct flow channel structure. (**d**) Small blades under gradient flow channel structure.

The dynamic pressure distribution on the impeller surfaces under different channel structures is presented in Figure 6. A comparative analysis of the simulation results reveals a decisively stronger machining potential in the gradual flow channel compared to the direct flow channel.

In the direct flow channel structure, the dynamic pressure across the vast majority of the large and small blade surfaces remained very low. A noticeable pressure concentration was observed only at the blade trailing edges, while the values over the main body of the blades were negligible. This indicates that effective machining is confined to a very limited area.

Conversely, the gradual flow channel structure yielded a substantial increase in dynamic pressure across the entire impeller. The dynamic pressure on the large blade suction and pressure surfaces was elevated by approximately one order of magnitude. The distribution, however, was non-uniform, characterized by distinct high-pressure zones at the upper and lower ends of the pressure surface and a prominent low-pressure region in the mid-span of the suction surface. A similar trend was observed on the small blades, where the pressure surface exhibited a marked, stepwise increase in dynamic pressure from the leading edge to the trailing edge, with values significantly surpassing those on the suction surface.

In summary, the overall dynamic pressure level in the graduated flow channel structure was markedly higher than in the direct flow channel structure. This confirms that the micro-cutting effect of the abrasive particles is substantially enhanced within the graduated flow channel, leading to a more effective machining process over a greater portion of the workpiece surface.

The dynamic pressure distribution on both sides of the large blade in the graduated flow channel structure is not uniform, with a region of low-pressure intensity at the top of the suction surface and in the middle of the leaf root. The dynamic pressure of the pressure surface shows the phenomenon of high pressure at the upper and lower ends, with low pressure in the middle part, and there are some low dynamic pressure areas on both sides of the middle-to-upper parts. Considering the small blade, both sides of the surface dynamic pressure distribution show large differences, and the dynamic pressure of the suction surface shows an increasing trend from the middle region of the leaf to the outer side. The dynamic pressure at the pressure surface gradually increases from the base part of the leaf root to the surrounding region, and it is clearly higher than the dynamic pressure at the suction surface. Because the gradient flow channel structure is compact, space is reduced, and the chances of abrasive particles and workpiece surface collision friction increase, enhancing the workpiece processing effect. According to the results of the above analysis, the overall dynamic pressure level of the graduated flow channel structure is higher than that of the direct flow channel structure, showing that the micro-cutting effect of the abrasive particles is greater in the graduated flow channel structure, with the result that the abrasive flow has the more effective processing effect on the workpiece surface.

#### 4.1.2. Analysis of Impeller Surface Wall Shear Under Different Flow Path Structures

From the analysis of the flow channel dynamic pressure characteristics, it can be seen that the graduated flow channel structure can improve the processing effect of the abrasive flow polishing fluid on the impeller workpiece. However, the wall shear is selected for analysis in this subsection in order to further determine the effect of the graduated flow channel structure on the processing effect. Wall shear is the slip force generated on the contact surface when the abrasive flow processes the surface of the workpiece, and it is an important indicator of the abrasive stream’s effectiveness on the workpiece.

**Figure 7:**
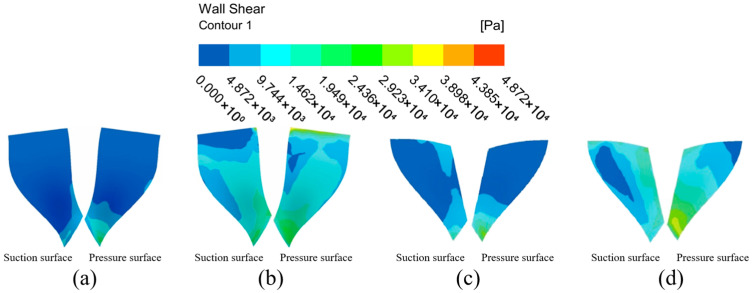
Cloud of wall shear distribution with different runner configurations. (**a**) Large blades under direct flow channel structure. (**b**) Large blades under gradient flow channel structure. (**c**) Small blades under direct flow channel structure. (**d**) Small blades under gradient flow channel structure.

The wall shear stress distribution on the large and small blades is analyzed in Figure 7 to further evaluate the processing effectiveness. Under the direct flow channel structure, the wall shear stress was generally low across all blade surfaces. A slight increase was detected only at the lower ends of the blades, particularly on the pressure surface of the large blade. This is attributed to the compressed fluid action space near the outlet. In contrast, the gradual flow channel structure dramatically enhanced the wall shear stress. The magnitude of wall shear on both the suction and pressure surfaces of the large and small blades increased significantly. The distribution on the large blade pressure surface showed a pattern of high values at the inlet and outlet and lower values in the middle. For the small blades, the wall shear on the pressure surface was consistently and substantially higher than on the suction surface, demonstrating a clear and progressive increase from the top to the bottom of the blade. Critically, the wall shear stress in the gradual channel was universally and significantly greater than that in the direct channel. This provides conclusive evidence that the graduated flow channel structure markedly improves the finishing efficiency of the abrasive flow.

The general trend in wall shear of the large blade suction surface under the gradual flow channel structure shows a gradual increase from the top to the bottom of the blade, and the wall shear effect at bottom part is slightly higher. The pressure distribution of the surface wall shear force shows that it is greater at the upper and lower ends than in the middle position, while in the middle position it is greater than on the left and right sides, and the overall distribution trend shows first a decrease and then an increase. And both the pressure surface or suction surface are significantly larger than the wall shear force under the direct flow channel structure. The wall shear stress distribution differs significantly between the two sides of the small blade. It increases radially from the central suction region, whereas the pressure surface shows a distinct vertical gradient from top to bottom, with substantially greater magnitudes. Because of the compact space between the gradual flow channel structure and the impeller, the abrasive grain movement is intensified, the kinetic energy of the particles increases, and the wall shear effect is improved. Although the impeller surface wall shear in the gradual flow channel is stratified, the difference in values between adjacent parts is within an order of magnitude, and it is significantly greater than the impeller wall shear in a direct flow channel.

In summary, the dynamic pressure and wall shear on the impeller surface is significantly higher in the graduated flow channel structure than in the direct flow channel structure, so the graduated flow channel structure was chosen for the numerical simulation of the abrasive flow processing parameters.

### 4.2. Numerical Analysis of the High-Performance Impeller by Solid–Liquid Abrasive Particle Flow

Because the previous section of the study illustrated that the gradual flow channel structure has a better effect for the abrasive flow processing of high-performance impellers, this channel structure is chosen for the next numerical simulation analysis of high-performance impeller workpieces. Combined with the actual abrasive flow machining conditions and related research experience, we choose the inlet pressure, inlet velocity, and abrasive viscosity as the factors, and analyze the dynamic pressure of the flow field and the wall shear to investigate the effect of abrasive flow on the surface machining quality of high-performance impellers.

#### 4.2.1. Numerical Analysis of High-Performance Impeller Surfaces at Different Inlet Pressures

The pressure pump provides abrasive movement power in the actual abrasive flow machining process, working on the surface of the workpiece to be finished. This is because when the input pressure increases, so does the radial force Fn and the axial force Fa on the abrasive grains, resulting in the abrasive grains’ processing capability. As a result, it is essential to investigate the effect of various inlet pressures on the processing effect of high-performance impellers for abrasive flow polishing. An abrasive volume percentage of 40%, abrasive particle size of 80 μm, and abrasive viscosity of 0.5 Pa·s are used in this study for the numerical simulation analysis of pressures.

(1) Dynamic pressure analysis of high-performance impeller surfaces at different inlet pressures.

**Figure 8:**
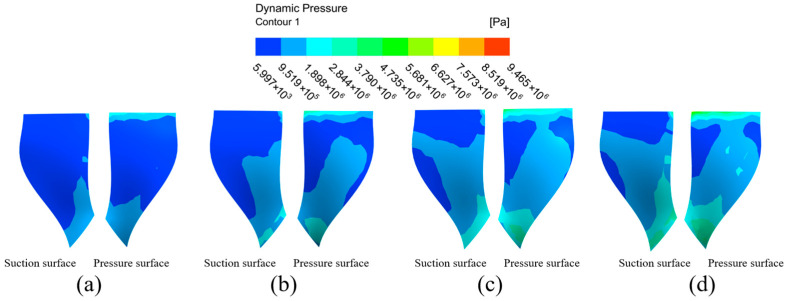
Cloud of dynamic pressure distribution of the large blade at different inlet pressures: (**a**) inlet pressure 3 MPa, (**b**) inlet pressure 4 MPa, (**c**) inlet pressure 5 MPa, and (**d**) inlet pressure 6 MPa.

Figure 8 illustrates the dynamic pressure distribution on the large blade surface at varying inlet pressures. As the inlet pressure increased from 3 MPa to 6 MPa, the area of low dynamic pressure on both the suction and pressure surfaces was reduced by over 70%. The average dynamic pressure increased linearly with the inlet pressure, from 120 kPa at 3 MPa to 480 kPa at 6 MPa on the suction surface. Despite this overall improvement, localized low-pressure zones persisted, particularly at the top of the suction surface and the middle-to-upper area of the pressure surface, indicating these areas are more challenging to polish.

**Figure 9:**
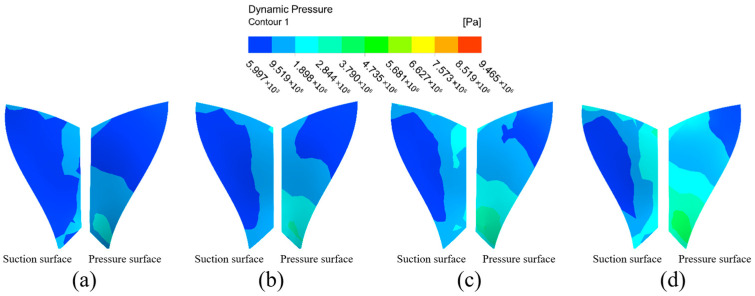
Cloud of dynamic pressure distribution for the small blade at different inlet pressures: (**a**) inlet pressure 3 MPa, (**b**) inlet pressure 4 MPa, (**c**) inlet pressure 5 MPa, and (**d**) inlet pressure 6 MPa.

Figure 9 shows the dynamic pressure distribution at each position after extracting both sides of the small blade surface for further analysis. With inlet pressure increases, the dynamic pressure intensity on the suction surface of the small blades increases gradually from the left center region to all around. The overall color is darker and the dynamic pressure intensity is lower when the inlet pressure is 3 MPa. When the input pressure is 6 MPa, the overall color of the blade becomes lighter from the left middle to all around, and the dynamic pressure intensity increases further, indicating that the processing effect on the suction surface’s outer edge will be better than in other sections. The dynamic pressure of the small blade’s pressure surface increases along the upper end of the blade root to the lower end of the blade. The color of the small blade’s pressure surface decreases gradually from the top to the bottom of the blade, while the dynamic pressure increases gradually in a stepwise ascending trend. This phenomenon becomes more visible with inlet pressure increases, showing that the bottom of the small blade has a better processing effect than other positions, and the whole surface processing effect is more uniform. At the same time, it can be concluded that the abrasive particle flow is more effective for pressure surface processing on the small blade.

**Figure 10:**
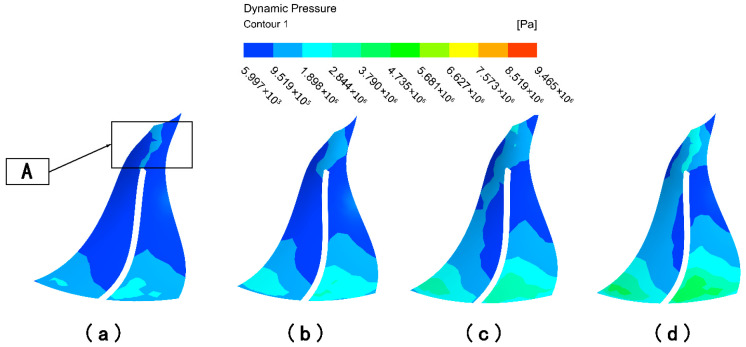
Cloud of dynamic pressure distribution at the hub surface of the impeller at different inlet pressures: (**a**) inlet pressure 3 MPa, (**b**) inlet pressure 4 MPa, (**c**) inlet pressure 5 MPa, and (**d**) inlet pressure 6 MPa.

Because the high-performance impeller is a complex curved structure, the dynamic pressure distribution in the cloud of large and small blades is not sufficiently comprehensive, and the impeller cannot be observed in some positions. Therefore, the surface of the impeller hub is extracted for further analysis, and the dynamic pressure distribution at each position is shown in Figure 10.

As shown in Figure 10, the color is lighter in the middle and darker on both sides of the impeller wheel face at position A, indicating that the dynamic pressure at the inlet of the runner is significantly greater at the center of the wheel than on the left and right sides. The dynamic pressure decreases from the middle position to the left and right sides in a stepwise distribution more obviously as the inlet pressure increases, which indicates that the processing effect of abrasive flow at the middle position is better than on the left and right sides at the face end of the hub. This is because the upper end of the small blade resists the fluid when it enters the flow channel, and the fluid and wall collisions cause the particle movement to become more irregular, improving the processing effect on the impeller wall surface processing. The dynamic pressure is similarly distributed in a stepwise way with the direction of fluid movement at the middle and lower positions of the hub surface. As the inlet pressure increases, the dynamic pressure at the intersection of the left half of the hub surface and the small blade suction surface is also increased. In summary, the increasing inlet pressure not only increases the dynamic pressure of the hub surface, but also improves the polishing uniformity.

(2) Wall shear analysis of high-performance impeller surfaces at different inlet pressures.

**Figure 11:**
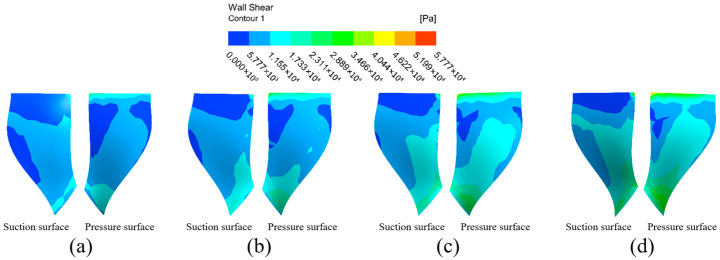
Cloud of wall shear distribution of the large blade at different inlet pressures: (**a**) inlet pressure 3 MPa, (**b**) inlet pressure 4 MPa, (**c**) inlet pressure 5 MPa, and (**d**) inlet pressure 6 MPa.

The wall shear distribution of the impeller at different inlet pressures is shown in Figure 11. The wall shear distribution on the large blade surface shows the same trend at different inlet pressures. The wall shear in the top and left regions of the large blade suction surface is less than the rest of the surface, indicating that the machining effect is better at the impeller’s edge, and that the machining effect of the abrasive flow on the impeller improves along the root of the blade to the blade’s edge. The wall shear in the upper-middle parts of the left and right sides of the pressure surface of the large blade is small, while the wall shear in the upper inlet and lower outlet is large, and the general distribution decreases first and increases second. With the inlet pressure gradually increased to 6 MPa, the wall shear on the suction surface at the top of the large blade increases slightly, and the low wall shear region in middle of the blade on the left essentially disappears. The low wall shear region at the pressure surface’s leaf root vanishes, and wall shear at the blade’s edge increases significantly.

**Figure 12:**
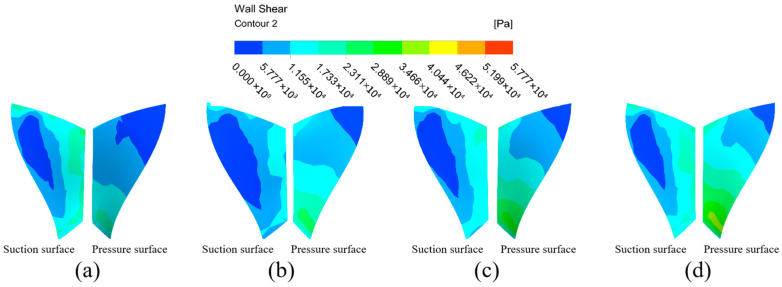
Cloud of wall shear distribution of the small blade at different inlet pressures: (**a**) inlet pressure 3 MPa, (**b**) inlet pressure 4 MPa, (**c**) inlet pressure 5 MPa, and (**d**) inlet pressure 6 MPa.

The wall shear distribution cloud of the small blade is shown in Figure 12. The wall shear on the suction surface of the small blade increases radially from the left side of the blade to the outer edge of the blade. The pressure surface wall shear from the upper end of the blade to the lower end of the blade shows a stepwise increasing distribution. The distribution trend of the shear force on both sides of the small blade becomes more obvious with the inlet pressure increases, and the wall shear on the pressure surface is obviously greater than that on the suction surface, indicating that the abrasive flow has a better processing effect on the right half and the lower end of the suction surface, and a better processing effect on the pressure surface as a whole.

**Figure 13:**
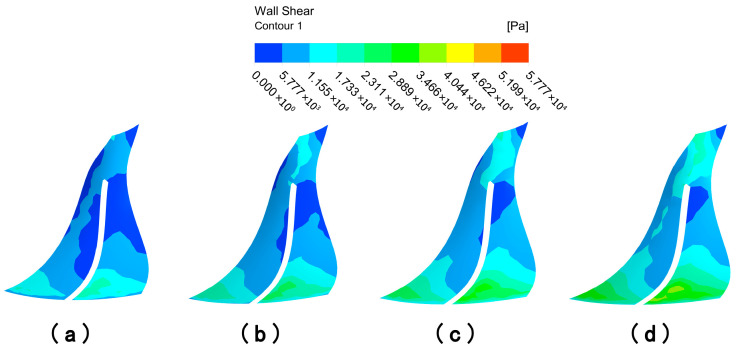
Cloud of wall shear distribution at the hub surface of the impeller at different inlet pressures: (**a**) inlet pressure 3 MPa, (**b**) inlet pressure 4 MPa, (**c**) inlet pressure 5 MPa, and (**d**) inlet pressure 6 MPa.

Figure 13 depicts the extraction of the impeller hub surface for analysis. The wall shear at the middle position of the hub’s surface top end is greater than the surrounding small region. This shows that the collision between the fluid and the leading edge of the small blade at the face end of the hub intensifies the particle motion, making the abrasive flow more effective for the intermediate position. There is a low shear region where the wheel face intersects the small blades, but this phenomenon is mitigated with the increase in inlet pressure. When the inlet pressure is 3 MPa, the wheel face has a low wall shear region on both the left and right sides. When the inlet pressure increases to 6 MPa, the low wall shear region on the left side of the wheel face virtually vanishes, while the low shear region on the right side decreases significantly, and the overall wall shear uniformity and processing effect on the wheel face improve.

The flow channel model of the high-performance impeller is complex, and the blade has a certain curvature shape. In the abrasive flow machining process, there is vortex formation between the large blade suction surface and small blade pressure surface, so the small blade suction surface pressure, from the root of the leaf to the outer edge, shows a stepwise distribution. There is a direct current between the large blade suction surface and small blade pressure surface, so the small blade suction surface pressure shows an increasing trend from top to bottom. In summary, increasing the inlet pressure will significantly improve the processing effect of abrasive flow on the workpiece. However, too much pressure will not only waste resources, but will also create the transition polishing phenomena. This is also consistent with the scientific studies of Li and Sui, et al. and strengthens this article’s scientific rigor [27].

#### 4.2.2. Numerical Analysis on the Surface of the High-Performance Impellers at Various Inlet Speeds

The abrasive grain flows alongside the base transportation fluid flow during the abrasive grain flow machining process. The initial velocity attained by the abrasive grain increases as the inlet velocity increases, as does the kinetic energy and the abrasive grain’s capacity to collide with the wall surface. As a result, it is equally essential to investigate the influence of abrasive inlet velocity on the processing effect. An abrasive volume percentage of 40%, abrasive particle size of 80 μm, and abrasive viscosity of 0.5 Pa·s are used in this study for the numerical simulation analysis of speed.

(1) Dynamic pressure analysis of high-performance impeller surfaces at different inlet speeds.

**Figure 14:**
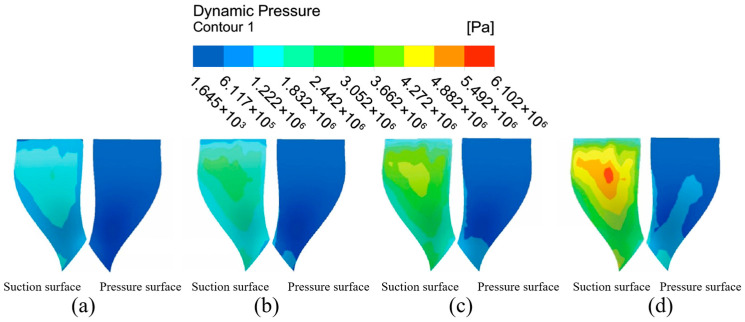
Cloud of dynamic pressure distribution of large blades at different inlet velocities: (**a**) inlet velocity 25 m/s, (**b**) inlet velocity 30 m/s, (**c**) inlet velocity 35 m/s, and (**d**) inlet velocity 40 m/s.

The dynamic pressure distribution on the large blade’s surface at different inlet speeds is displayed in Figure 14. With an increase in inlet speed, the dynamic pressure on the suction surface of the large blade intensified markedly within the central region, from which it decreased radially towards the periphery. This resulted in a pronounced stratification of the dynamic pressure distribution. In contrast, the dynamic pressure on the pressure surface remained comparatively low throughout, with its magnitude being substantially lower—by more than an order of magnitude in many areas—than that on the suction surface. This indicates a significant disparity in the machining effectiveness between the two sides of the large blade. The observed enhancement in dynamic pressure with increasing inlet speed confirms that the kinetic energy of the abrasive particles and their consequent machining effect on the blade surface are strengthened.

**Figure 15:**
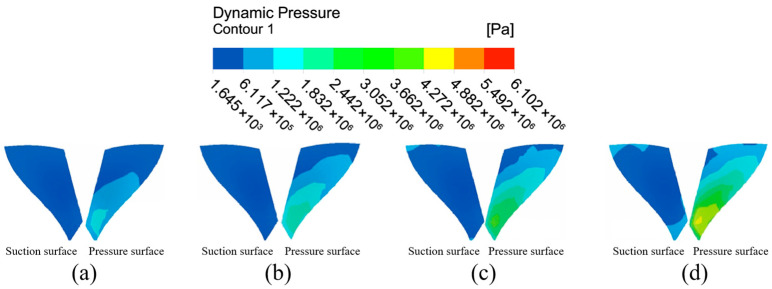
Cloud of dynamic pressure distribution of small blades at different inlet velocities: (**a**) inlet velocity 25 m/s, (**b**) inlet velocity 30 m/s, (**c**) inlet velocity 35 m/s, and (**d**) inlet velocity 40 m/s.

Observation of the small blade surfaces in Figure 15 reveals a distribution pattern opposite to that of the large blade. The dynamic pressure on the suction surface of the small blade was generally low and showed little significant variation with increasing speed. Conversely, the pressure surface exhibited a clear trend of increasing dynamic pressure from the leading edge to the trailing edge. This gradient became more defined at higher inlet speeds, demonstrating that the processing effect on the small blade pressure surface is not only stronger than on its suction surface but also intensifies notably with increased flow velocity.

**Figure 16:**
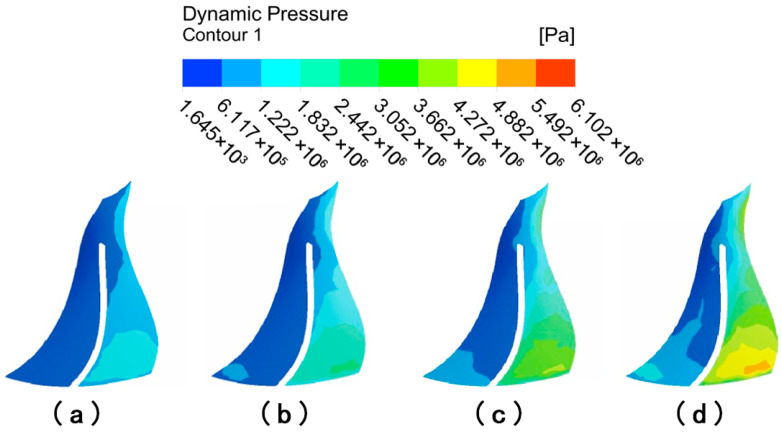
Cloud of dynamic pressure distribution at the hub surface of the impeller at different inlet velocities: (**a**) inlet velocity 25 m/s, (**b**) inlet velocity 30 m/s, (**c**) inlet velocity 35 m/s, and (**d**) inlet velocity 40 m/s.

Figure 16 shows the extraction of the impeller’s hub surface for inspection. There are large differences in dynamic pressure distribution between the left and right parts of the hub surface. The dynamic pressure on the left half of the wheel is lower and does not change significantly from the top to the bottom of the wheel, while the dynamic pressure on the right half is obviously greater, gradually increasing from the top to the bottom end of the wheel. As a result, the abrasive flow polishing fluid processing effect on the right half of the hub surface is better than on the left half. With increasing speed, the kinetic energy of the fluid increases, the effect of the particles on the workpiece surface becomes more pronounced, and the dynamic pressure on the hub surface increases continuously. However, the increase in dynamic pressure on the left half is smaller, while the increase on the right half is greater.

(2) Wall shear analysis of high-performance impeller surfaces at different inlet speeds.

**Figure 17:**
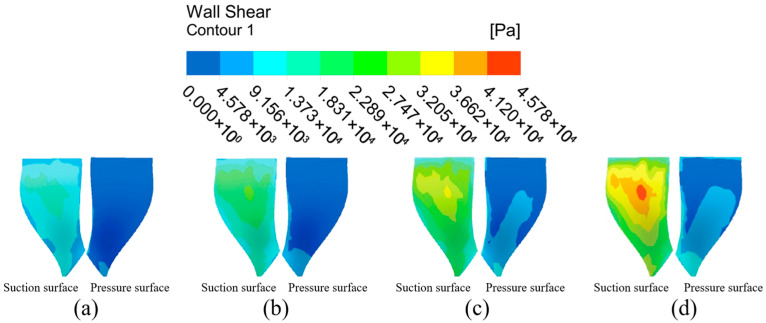
Cloud of wall shear distribution of large blades at different inlet velocities: (**a**) inlet velocity 25 m/s, (**b**) inlet velocity 30 m/s, (**c**) inlet velocity 35 m/s, and (**d**) inlet velocity 40 m/s.

The wall shear distribution on the large blade at various inlet speeds is depicted in Figure 17. The wall shear on the suction surface mirrored the distribution trend of the dynamic pressure, with the highest values concentrated in a central zone that decreased radially. This pattern became more distinct and the magnitude of the shear stress increased considerably with higher inlet velocities. The wall shear on the pressure surface was significantly lower in comparison. While an increase in inlet speed to the highest levels did lead to a moderate improvement in wall shear at the middle-to-lower sections of the pressure surface, the processing effectiveness on the suction surface remained substantially superior.

**Figure 18:**
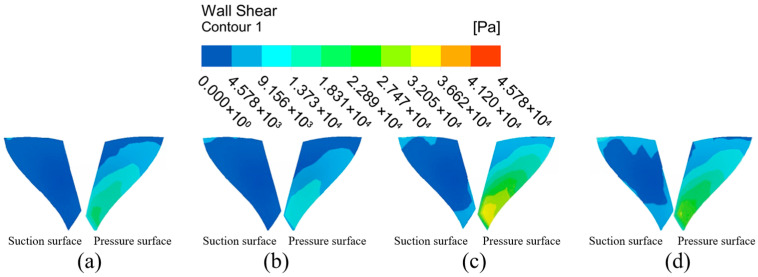
Cloud of wall shear distribution of small blades at different inlet velocities: (**a**) inlet velocity 25 m/s, (**b**) inlet velocity 30 m/s, (**c**) inlet velocity 35 m/s, and (**d**) inlet velocity 40 m/s.

Figure 18 shows the wall shear distribution for the small blades. The wall shear on the suction surface was generally low and uniform. At the highest inlet speed, a noticeable increase in shear stress was observed at the inlet and outlet edges, but the overall effect remained limited. In stark contrast, the pressure surface exhibited a well-defined wall shear distribution that increased progressively from the leading edge to the trailing edge. This gradient intensified markedly with increasing inlet speed, unequivocally demonstrating that the abrasive flow acts more effectively on the pressure surface, particularly towards the trailing edge.

**Figure 19:**
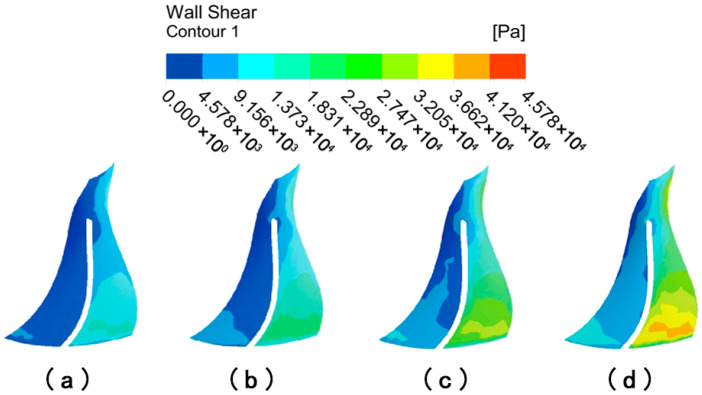
Cloud of wall shear distribution at the hub surface of the impeller at different inlet velocities: (**a**) inlet velocity 25 m/s, (**b**) inlet velocity 30 m/s, (**c**) inlet velocity 35 m/s, and (**d**) inlet velocity 40 m/s.

The extracted wall shear on the hub surface is shown in Figure 19. The shear stress on the left half of the hub was significantly weaker than on the right half. As the inlet velocity increased, the wall shear on the left half showed some improvement, but the processing uniformity remained poor. The right half sustained a much higher level of wall shear, which increased steadily from the flow inlet to the outlet. The increase in abrasive movement speed significantly enhanced the micro-cutting effect on the impeller wall; however, it also led to a severe non-uniformity in the processing effect across different regions of the hub.

Abrasive polishing fluid flows at certain speeds in a straight-line direction into the flow channel, colliding directly with the top of the small blade. This changes the fluid direction, and the particles’ kinetic energy is enhanced. Increased force in the middle of the large blade’s suction surface results in an uneven processing effect. Because the blade shape is more complex and has a certain curved shape, the direction of abrasive movement is disorganized. As the blade force position changes, the small blade pressure surface force increases, generating a non-uniform effect. The kinetic energy of the particles increases with increased inlet speed, and the cutting effect improves; however, excessively fast speeds aggravate the uneven processing effect on both sides of the blade, impairing the entire processing effect.

#### 4.2.3. Numerical Analysis on the Surface of High-Performance Impellers at Various Abrasive Viscosities

Abrasive viscosity represents the interaction between the liquid and the particles, and the level of the abrasive viscosity can affect the collision efficiency between the particles and the wall, influencing the machining quality of the workpiece, so analyzing the impact of the abrasive viscosity on the machining effect is also very important. An inlet pressure of 5 MPa and abrasive particle size of 80 μm are used in this study for the numerical simulation analysis of abrasive viscosity.

**Figure 20:**
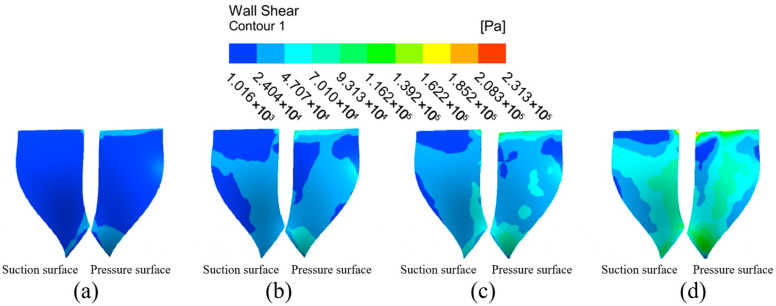
Cloud of wall shear distribution of large blades at different abrasive viscosities: (**a**) abrasive viscosity 0.1 Pa·s, (**b**) abrasive viscosity 0.3 Pa·s, (**c**) abrasive viscosity 0.5 Pa·s, and (**d**) abrasive viscosity 1 Pa·s.

Figure 20 depicts the distribution of wall shear on the surface of large blades with different abrasive viscosities. When the abrasive viscosity is 0.1 Pa·s, the overall color of the suction and pressure surfaces of the large blade is darker, while the color at the lower end of the large blade is slightly lighter, indicating that the overall wall shear of the large blade is small and that the wall shear at the outlet is only slightly increased. When the abrasive viscosity is 0.3 Pa·s, the color of the left and upper regions of the large blade’s suction surface is darker, as is the color of the area on both sides of the pressure surface, indicating that wall shear in the middle area of the large blade increases and the machining effect improves. When the abrasive viscosity increases to 0.5 Pa·s or 0.1 Pa·s, the wall shear at the upper end of the suction surface of the large blade remains small, and the dark color of the left area of the suction surface, i.e., the position of the suction surface leaf root, vanishes and becomes lighter in color, indicating that the wall shear at this location is obviously larger, and the overall uniformity improves. The deeper color of the pressure surface’s low wall shear region gradually decreases, showing that increasing the viscosity of the abrasive grains increases the processing effect of the abrasive grain flow.

**Figure 21:**
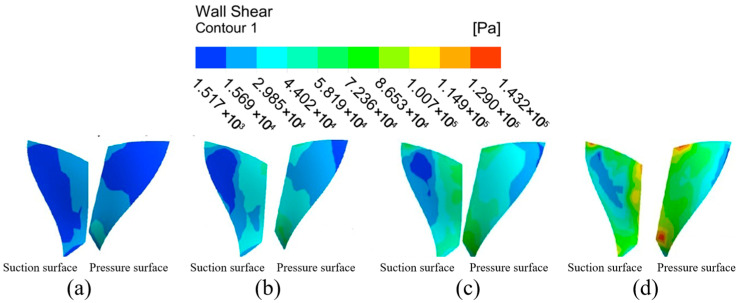
Cloud of wall shear distribution of small blades at different abrasive viscosities: (**a**) abrasive viscosity 0.1 Pa·s, (**b**) abrasive viscosity 0.3 Pa·s, (**c**) abrasive viscosity 0.5 Pa·s, and (**d**) abrasive viscosity 1 Pa·s.

Figure 21 depicts the distribution of wall shear on the surface of small blades with different abrasive viscosities. When the viscosity is 0.1 Pa·s, the left region of the small blade’s suction surface is darker, with less wall shear at the root of the blade, while the top middle region of the small blade’s pressure surface is darker, with less wall shear at the top of the blade. As the abrasive viscosity increases, the wall shear of the abrasive flow on the surface of the small blades becomes more uniform. The suction surface of the small blade’s wall shear is radial in the region of the left side of the blade and gradually increases in all directions; the pressure surface’s wall shear is centered in the upper section of the blade and distributed in a stepwise incremental manner in all directions. The darker colored low-shear areas further decrease, suggesting that increased abrasive viscosity has an overall positive influence on the processing of small blades.

**Figure 22:**
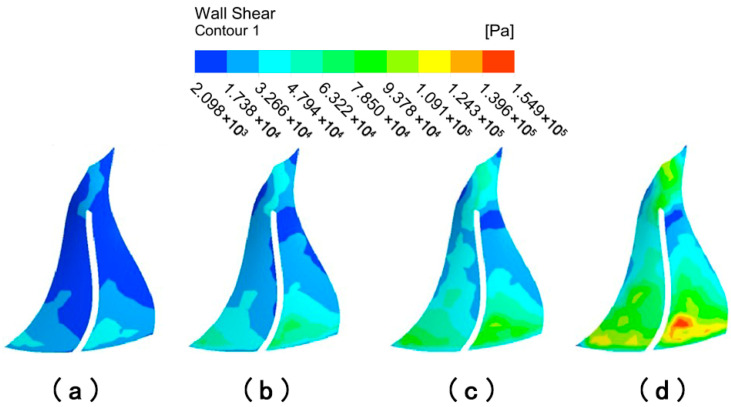
Cloud of wall shear distribution at the hub surface of the impeller at different abrasive viscosities: (**a**) abrasive viscosity 0.1 Pa·s, (**b**) abrasive viscosity 0.3 Pa·s, (**c**) abrasive viscosity 0.5 Pa·s, and (**d**) abrasive viscosity 1 Pa·s.

The impeller wheel surface was extracted for further analysis to determine the effect of abrasive particle flow viscosity on the machining effect of different impeller structure surfaces, as shown in Figure 22. The wall shear from the middle to both sides decreases in the wheel face end position. This is due to the fluid in the upper end of the small blade encountering resistance to movement becoming turbulent, with no resistance to movement on both sides, continuing in the original direction of movement. The wall shear at the wheel’s face end is greater than the wall shear on both sides. It is also discovered that the wall shear increases gradually from the middle to the lower end of the wheel face. There is no significant difference in the magnitude of the wall shear between the blade and right sides of the faces, which are reasonably uniformly distributed. The overall wall shear force increases as the viscosity gradually increases from 0.1 Pa·s to 1 Pa·s. This indicates that increasing the inlet viscosity improves the abrasive flow’s effect on the workpiece surface.

The particles move under the covering of the liquid phase during the process of abrasive particle flow movement. When the fluid viscosity is low, the fluid in the narrow and complex curved flow channel moves chaotically and irregularly, while the fluid collides with the wall after the turbulence phenomenon intensifies, and the particle action on the wall becomes haphazard and irregular. When the viscosity of the fluid is high, the collision of the fluid produces vortices. As the viscosity is restricted, the probability of a large vortex breaking into small vortices and energy collapse decreases, and the processing effect is enhanced. However, excessive viscosity of the abrasive particles will increase fluid flow resistance, decreasing processing efficiency [28]. In summary, when the viscosity is 0.5 Pa·s, it not only produces a superior processing effect on the surface of the workpiece, but also saves cost and ensures fluid flow in the complex curved surface structure.

## 5. Experimental Processing and Results Analysis

In order to demonstrate the effectiveness of two-phase solid–liquid abrasive particle flow for precise machining of high-performance impeller surfaces, numerical simulation analysis and actual precise processing conditions of abrasive flow were combined. Three process factors, namely, inlet pressure, processing times and abrasive grain size, were selected as the objectives of the study. The inlet pressures were 4 MPa, 5 MPa and 6 MPa, the number of operations was 10, 20 and 30, and the grain sizes were 40 μm, 80 μm and 120 μm, corresponding to fine, medium, and coarse grains. The control table of orthogonal test settings for a three-factor, three-level orthogonal test with nine groups is shown in Table 2.

Solid–liquid two-phase abrasive flow machining tests were carried out on high-performance impeller workpieces according to the designed orthogonal test table. The test workpiece is the most frequently used high-performance integrated impeller workpiece in vehicle engines. The workpiece is made of aluminum alloy 2024 and complies with the national standard 06Cr19Ni10, as for the workpiece used in the simulation process, which enables a comparative experiment. The test parts are labeled A-01 through A-09, while the original piece is labeled A-00. The workpiece was cut to facilitate observation of the effect of abrasive flow machining on the surface of the high-performance impeller for different combinations of process parameters. The DK7732 EDM wire cutting machine (Taizhou Huazheng CNC Machine Tool Co., Ltd., Taizhou, China) was selected to cut the high-performance impeller workpiece, as well as cleaning and testing of workpiece parts.

### 5.1. Experimental Methodology and Characterization Techniques

To systematically evaluate the surface quality of the high-performance impellers after abrasive flow machining, the following characterization techniques were employed on the sectioned workpiece samples:

Laser confocal microscopy (LCM): The three-dimensional surface morphology and topography of the samples were examined using a ZEISS LSM700 laser confocal microscope (Carl Zeiss AG, Oberkochen, Germany). This technique was used to qualitatively assess the removal of burrs, the reduction in scratches, and the overall smoothing of the surface at specific locations on the large and small blades. Scanning electron microscopy (SEM): The microscopic surface morphology of the blade’s suction surface was characterized using a German Zeiss EVO MA25 scanning electron microscope(Carl Zeiss AG, Oberkochen, Germany). The observations were conducted at a standard magnification of 60× to precisely analyze the removal of micro-scale burrs, the evolution of surface scratches, and the presence of any pitting or defects. Surface roughness stylus profilometry: The arithmetic mean roughness (Ra) of the impeller blade surfaces was quantitatively measured using a Mahr LD120 surface roughness stylus (Mal Group, Göttingen, Germany). To ensure data accuracy and consistency, a stylus travel distance of 10 mm was selected for all measurements, which were performed on the middle position of the blade surfaces. A coordinate measuring machine (CMM) or high-precision optical profiler was used to measure the following characteristics of the blade before and after processing: blade thickness—multiple sections at the leading edge, trailing edge, and middle of the blade were selected for measurement of thickness changes; leading/trailing edge fillet radius—the fillet radius of the leading and trailing edges that are prone to polishing were accurately measured; and contour accuracy—the processed blade contour was compared with the original CAD model to evaluate its contour deviation.

### 5.2. Laser Confocal Microscopy Inspection and Analysis

The influence of different processing conditions on the finishing effectiveness was assessed by examining the three-dimensional morphology of the sample workpieces using laser confocal microscopy (as described in Section 5.1).

**Figure 23:**
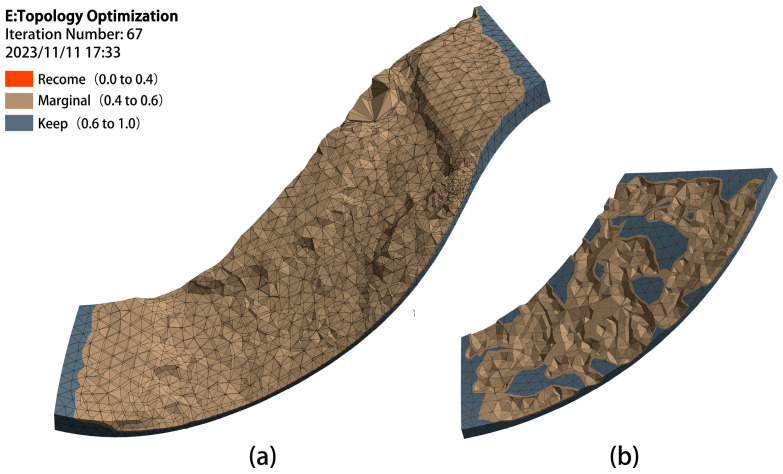
The Blade topology optimization analysis diagram (**a**) the large Blade. (**b**) the small Blade As illustrated in Figure 23, the Z-axis represents the height of surface detection, the highest is the red surface, followed by yellow, green and blue, respectively.

Surface topology optimization simulations of the large and small impellers were performed to identify regions susceptible to weaker processing effects, consistent with the numerical simulation analysis, as illustrated in Figure 23. The middle region of the large blade’s pressure surface and the small blade’s suction surface, identified as such, were selected for wire-cutting and subsequent surface inspection to determine the effectiveness of abrasive flow polishing.

**Figure 24:**
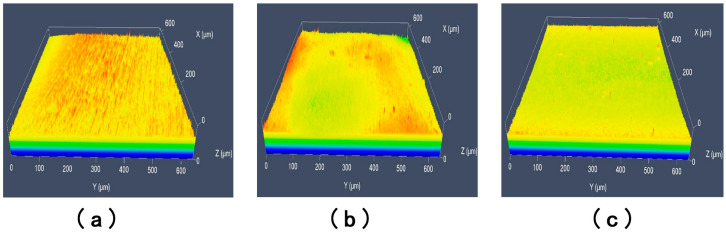
Laser confocal microscope inspection diagrams for the large blades (**a**) A-03, (**b**) A-06, and (**c**) A-09. The color gradient (from blue to yellow/red) corresponds to the vertical height (Z-axis) of the surface: blue represents the lowest regions (near 0 μm), while yellow/red indicates the highest regions (up to ~2 μm).

As shown in Figure 24a, the three-dimensional morphology of the surface of sample A-03 shows that the dark red color of the surface has almost completely disappeared, but there is still a wide region of light red color. This clearly illustrates that the unevenness of the sample’s surface has been decreased and a large number of bumps have been eliminated, but the surface scratches and bumps have not completely disappeared. Figure 24b depicts the significant improvement in the surface profile in the morphology of sample A-06. The surface burr has almost completely disappeared, the number of scratches has decreased, the surface has flattened, and the surface quality has improved. However, because the surface of the blade is curved, there are still burrs that have not been removed. The Figure 24c indicates that surface burrs and surface scratches on the morphology of sample A-09 have disappeared, resulting in a relatively smooth surface, proving the effectiveness of abrasive flow cleaning of the impeller surface.

**Figure 25:**
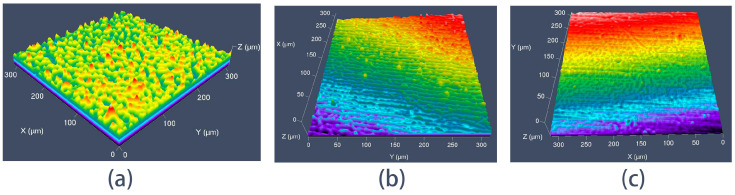
Laser confocal microscope inspection diagrams for the small blades (**a**) A-00, (**b**) A-07, and (**c**) A-09. The color gradient (from blue to red/yellow) corresponds to the vertical height (Z-axis) of the surface: blue denotes the lowest regions, while red/yellow indicates the highest regions.

To further demonstrate the homogeneity of the abrasive flow machining, the laser confocal microscope was used to inspect the middle position of the suction surfaces of the small wire-cut blades A-00, A-07, and A-08, and the results are shown in Figure 25. Figure 25a indicates that the original A-00 sample with the surface that was not processed by abrasive grain flow has a huge number of burrs, which severely restricts the workpiece’s application. Figure 25b indicates that enormous numbers of burrs have been removed, with only a few bumps and scratches remaining. Figure 25c illustrates that the surface burrs have completely disappeared, resulting in the rather smooth surface, demonstrating the efficiency of abrasive flow machining.

According to the three-dimensional surface morphology analysis, it can be concluded that under the same conditions of processing time, increasing the inlet pressure can increase the kinetic energy of the abrasive particles themselves, and increasing the abrasive particle size can increase the probability of the abrasive particles touching the workpiece, both of which effectively improve the effectiveness of the abrasive particle flow in uniformly processing the surface and improve the surface quality of the impeller.

### 5.3. Scanning Electron Microscope Inspection and Analysis

**Figure 26:**
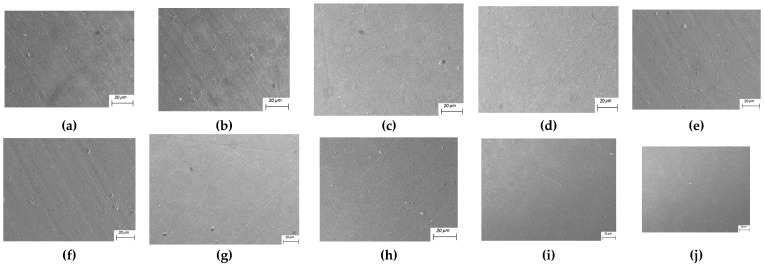
Scanning electron microscopy (SEM) images showing the surface morphology of the blade’s suction surface under different processing parameters: (**a**) unprocessed original workpiece (A-00), (**b**) A-01, (**c**) A-02, (**d**) A-03, (**e**) A-04, (**f**) A-05, (**g**) A-06, (**h**) A-07, (**i**) A-08, and (**j**) A-09. All images were acquired at an accelerating voltage of 15 kV using a secondary electron detector.

As shown in Figure 26, the original A-00 sample has numerous irregular scratches, agglomerated burrs, deep scratches, a rough surface, and poor surface quality. The surface morphology of sample A-01 shows that the number of burrs on the surface has been greatly reduced and the depth of scratches has become shallower, but there are still more small bumps on the surface and scratches are visible. The surface of sample A-02 is clearly scratched, and although the surface quality has improved significantly, there are still pitting and pits around the scratches. The number of scratches on the surface of sample A-03 has been reduced, and the surface uniformity has improved, but there are still wider and deeper scratches, and burr accumulation is still serious. Sample A-04 has no obvious scratches on the surface except for at the edges, and the agglomerated burrs have disappeared, but there are still a few dots with irregularities on the surface. Sample A-05 has a larger number of burrs than sample A-04, but no visible scratches on the surface. The surface of sample A-06 has a large number of burrs removed, but there are pits and sharp scratches. This is caused by a high number of surface processes and particles striking the surface of the workpiece multiple times, which can be corrected by adjusting the number of processes. The surface of sample A-07 is flat and smooth, and the burrs and bumps have disappeared. However, the small mesh size and large particle size under this processing condition also cause pits and shallow scratches on the surface of the workpiece. The surface of sample A-08 is smooth, the scratch marks have disappeared, there is no burr protrusion, the surface quality is better, and the surface quality has significantly improved. The surface shape of sample A-09 has become smooth and flat, but there are still scratches on the surface. According to the above analysis, the burrs and pits on the workpiece’s surface are basically removed after the finishing process by the abrasive flow, and the surface quality is significantly improved, demonstrating the effectiveness of the abrasive flow process.

### 5.4. Surface Roughness Stylus Inspection and Analysis

**Figure 27:**
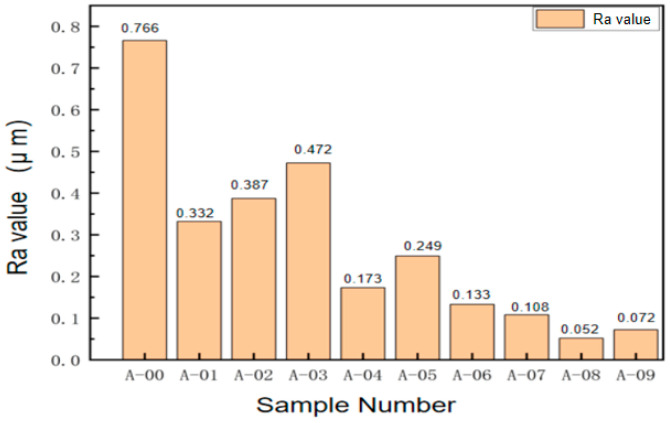
Impeller blade surface roughness variation.

To accurately evaluate the machining effect of different process parameters on the overall impeller, the surface roughness of the middle position of the blade was quantitatively measured. As shown in Figure 27, the surface roughness of the untreated workpiece (A-00) was the highest, reaching 0.766 μm. After abrasive flow polishing, the surface roughness of each specimen decreased to varying degrees. When the inlet pressure was 5 MPa and the sample was processed 20 times with an abrasive particle size of 120 μm (A-05), the surface roughness decreased to 0.249 μm, a decrease of 67.49% from the initial value. By continuously optimizing process parameters, the best polishing effect was achieved on the A-08 specimen, with surface roughness reduced to 0.052 μm, an order of magnitude improvement.

The high-performance impeller surface roughness test data are shown in Table 3. The roughness variation graph is plotted with the different test specimens as horizontal coordinates and the surface roughness values as vertical coordinates. The histogram provides a visual comparison of the surface finish of the workpiece blade under different abrasive flow conditions. It can be seen that the surface roughness of the original workpiece is higher than 0.7 μm and that the surface quality is relatively poor. The surface roughness of each specimen is reduced to different degrees after polishing by the abrasive flow. When the pressure at the inflow port of abrasive grains is 5 MPa, the number of processing times is 20, and the abrasive grain size is 120 μm, the surface roughness of sample A-05 is reduced to 0.249 μm, which is 67.49% lower than the original surface roughness. When the processing parameters are continuously adjusted, the optimum processing of the blade surface by the abrasive flow can be improved by an order of magnitude. The surface roughness is reduced from 0.766 μm to 0.052 μm, and the abrasive grain flow processing effect is excellent. It was also found that, in abrasive flow processing, there should be an appropriate choice of processing parameters, otherwise the processing will damage the workpiece’s surface, resulting in the transition polishing phenomenon [29].

### 5.5. Analysis of Variance for Orthogonal Tests

In order to determine the effect of abrasive flow machining technology on the machining of high-performance impellers, the corresponding roughness data values were analyzed statistically using ANOVA and regression analysis methods. The analysis of variance (ANOVA) method is used to determine the degree of difference between data fluctuations produced by factors and data fluctuations caused by errors. The importance of the difference in overall averages at different levels of the associated factors is inferred, and factor interactions can also be identified. The results in this experiment are analyzed using analysis of variance (ANOVA), as shown in Table 4.

In the statistical significance test, the size of the *p*-value determines the significance of a test factor on the results; the smaller the *p*-value, the greater the significance of the test factor. According to ANOVA Table 3, the effect of inlet pressure on the test index is the most significant, followed by the influence of abrasive particle size, while the effect of the number of processes on the test index is not significant. It can be concluded that the three test factors in this test have the following order of influence on the overall impeller processing effect of the abrasive flow: inlet pressure > abrasive particle size > number of times processed.

**Figure 28:**
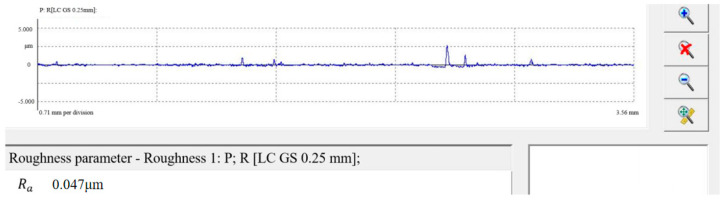
Surface roughness inspection values.

In order to further confirm the correctness of the data analysis and the effectiveness of the abrasive flow process, the best combination of elements to increase the surface finish of the impeller was investigated. Table 5 shows the mean response analysis, where the lower the value, the better the representative factor. For example, with inlet pressure, 6 MPa has the smallest value of 0.07733; therefore, 6 MP is the best factor. As a result, the perfect parameter combination for polishing high-performance impellers under these test conditions is an inlet pressure of 6 MPa, a process number of 10 times, and an abrasive grain size of 40 µm. This analysis also demonstrates that the processing effect of high-performance impellers is influenced in the following order: inlet pressure > abrasive grain size > number of processes. This is consistent with the preceding conclusions. To demonstrate the accuracy and rigorousness of the conclusions, an additional set of abrasive flow polishing tests were performed, and the roughness values of the polished surfaces were analyzed, as shown in Figure 28. Surface roughness was measured at 0.047 μm under these machining conditions, which is an improvement in surface quality compared to the 0.052 μm measured on sample A-08.

### 5.6. Regression Analysis of the Orthogonal Test

The quality of high-performance impellers machined by abrasive flow is predicted and controlled using regression analysis to develop a multiple non-linear regression equation for surface roughness. Assuming the functional relationship between the dependent variable y (surface roughness) and the independent variables $x_{1}$ (inlet pressure), $x_{2}$ (number of operations) and $x_{3}$ (grain size), the mathematical model can be defined as follows:

(7) $$y=b_{0}+b_{1}x_{1}+b_{2}x_{2}+b_{3}x_{3}+b_{4}x_{1}x_{2}+b_{5}x_{1}x_{3}+b_{6}x_{2}x_{3}+b_{7}x_{1}x_{2}x_{3}$$

Table 6 displays the non-linear regression analysis table calculated for the relevant data. It can be seen that the constant term in this model is $b_{0}=0.735$; the coefficient of the independent variable $x_{1}$ is $b_{1}=-0.0848$; the coefficient of the independent variable $x_{2}$ is $b_{2}=0.0087$; the coefficient of the independent variable $x_{3}$ is $b_{3}=-0.00274$; the coefficient of the independent variable ${x_{1}x}_{2}$ is $b_{4}=-0.00339$; the coefficient of the independent variable ${x_{1}x}_{3}$ is $b_{5}=0.000232$; and the coefficient of the independent variable ${x_{2}x}_{3}$ is $b_{6}=0.000125$. Therefore, this linear regression equation is as follows:

(8) $$\begin{array}{c} y=0.735-0.0848x_{1}+0.0087x_{2}-0.00274x_{3}-0.00339x_{1}x_{2}\\ +0.000232x_{1}x_{3}+{0.000125x}_{2}x_{3} \end{array}$$

The R-sq is recognized as goodness of fit, and a higher R-sq value indicates that the regression model data is a better fit. The adjusted R-sq value represents the regression model’s dependability. The closer the adjusted R-sq value is to the original R-sq value, the more reliable the regression model. The fact that the R-sq of 98.66% and the R-sq (adjusted) of 94.66% in Table 6 are close to each other indicates that the non-linear regression model is valid. To judge the accuracy of the non-linear regression equation, a prediction experiment was performed on the test data using the 31 quationn as shown in Table 7. It can be observed from the graph that the predicted values mostly overlap with the detected values, with a small range of fluctuations, demonstrating the accuracy and reliability of the non-linear regression equation.

## 6. Conclusions

This study systematically investigated the quality control of abrasive flow precision machining for high-performance impellers through a combined approach of numerical simulation and experimental verification. The key findings, supported by the data, are summarized as follows: compared with the direct flow channel structure, the gradient flow channel structure has a more compact structure, which improves the collision efficiency between the abrasive particles and the workpiece. Therefore, the gradient flow channel structure can significantly increase the dynamic pressure and wall shear on the impeller surface, thus improving the efficiency of abrasive flow processing.

(1) Flow channel structure: The graduated flow channel structure was demonstrated to be superior to the direct flow channel. It significantly enhances the machining efficiency by increasing the collision frequency between abrasives and the workpiece, resulting in a marked increase in the dynamic pressure and wall shear stress on the impeller surface.
(2) Influence of process parameters: Inlet pressure: Increasing the inlet pressure was the most influential factor, leading to a substantial improvement in the intensity and uniformity of the machining effect across the impeller surface.
(3) Inlet velocity: While a higher inlet velocity intensified the overall machining effect, it concurrently exacerbated the non-uniformity of material removal, particularly creating a significant disparity between the blade’s pressure and suction surfaces.
(4) Abrasive viscosity: An optimal abrasive viscosity (0.5 Pa·s in this study) was identified, which effectively improves the machining uniformity without excessively increasing flow resistance.
(5) Experimental optimization and validation: Orthogonal experiments and analysis of variance (ANOVA) quantitatively confirmed that inlet pressure has the most significant impact on surface roughness, followed by abrasive grain size, with the number of processing cycles having a less pronounced effect. The optimal parameter combination (6 MPa inlet pressure, 10 cycles, and 40 μm abrasive size) was determined and validated. Under these conditions, the surface roughness of the impeller was reduced from an initial Ra of 0.766 μm to a final Ra of 0.047 μm, achieving a high-quality, uniformly polished surface.

In conclusion, this research establishes a feasible and effective strategy for controlling the surface quality of complex components like high-performance impellers using solid–liquid two-phase abrasive flow machining, providing a scientific basis for its engineering application.

## Figures and Tables

**Table 1:** Grid parameter table.

Parameter	DC Channel			Gradient Flow Channel		
Unit quantity (10,000)	60,372	73,022	89,118	42,364	54,911	72,887
Number of nodes (10,000)	13,150	15,662	18,790	8.2000	10,507	13,760
Grid quality (≥0.5)	0.793	0.797	0.802	0.838	0.840	0.841
Jacobi (<5)	2.221	2.319	2.150	1.845	1.838	1.834
Average skewness	0.201	0.201	0.199	0.222	0.219	0.218
Maximum skewness (<0.98)	0.850	0.852	0.851	0.810	0.840	0.832

**Table 2:** Comparison table of orthogonal test parameters for integral impellers.

Sample Number	Inlet Pressure (MPa)	Number of Processes (Times)	Grit Size (μm)
A-01	4	10	40
A-02	4	20	80
A-03	4	30	120
A-04	5	10	80
A-05	5	20	120
A-06	5	30	40
A-07	6	10	120
A-08	6	20	40
A-09	6	30	80

**Table 3:** Impeller surface roughness test values.

Number	A-00	A-01	A-02	A-03	A-04	A-05	A-06	A-07	A-08	A-09
Ra value (μm)	0.766	0.332	0.387	0.472	0.173	0.249	0.133	0.108	0.052	0.072

**Table 4:** Analysis of variance table.

Source	Freedom	AdjSS	AdjMS	F-Value	*p*-Value
Inlet pressure	2	0.156073	0.078036	148.83	0.007
Number of processes	2	0.001533	0.000766	1.46	0.406
Particle size	2	0.017594	0.008797	16.78	0.056
Error	2	0.001049	0.000524		
Total	8	0.176248			
S = 0.0228983		R-sq = 99.41%		R-sq (adjusted) = 97.62%	

**Table 5:** Mean response table.

Numbers	Inlet Pressure		Number of Processes		Particle Size	
	Factor	Value	Factor	Value	Factor	Value
1	4 MPa	0.39367	10 times	0.20100	40 μm	0.16900
2	5 MPa	0.18500	20 times	0.22933	80 μm	0.21067
3	6 MPa	0.07733	30 times	0.22567	120 μm	0.27633
Delta	0.31633		0.02833		0.10733	
ranking	1		3		2	

**Table 6:** Multiple non-linear regression analysis table.

Item	Coefficient	Standard Deviation of Coefficient	T-Value	*p*-Value	Variance Inflation Factor
Constants	0.735	0.252	2.92	0.100	
Inlet pressure	−0.0848	0.0560	−1.51	0.269	16.00
Number of processes	0.0087	0.0135	0.64	0.586	93.14
Abrasive grain size	−0.00274	0.00547	−0.50	0.666	244.00
Inlet pressure × Number of processes	−0.00339	0.00318	−1.07	0.398	152.57
Inlet pressure × Abrasive grain size	0.000232	0.000794	0.29	0.798	152.57
Number of processes × Abrasive grain size	0.000125	0.000079	1.57	0.257	44.57
S = 0.0343019		R-sq = 98.66%		R-sq (Adjusted) = 94.66%	

**Table 7:** Predicted values of roughness under non-linear regression.

No.	Detected Value	Predicted Value	Standardised Residuals	95% Confidence Interval	95% Prediction Interval
01#	0.324	0.325	0.033753	(0.179582, 0.470037)	(0.117750, 0.531869)
02#	0.385	0.354	0.024062	(0.250042, 0.457101)	(0.173291, 0.533852)
03#	0.475	0.482	0.033753	(0.336820, 0.627275)	(0.274988, 0.689107)
04#	0.174	0.202	0.024061	(0.098661, 0.305720)	(0.021910, 0.382471)
05#	0.253	0.256	0.024061	(0.152708, 0.359768)	(0.075958, 0.436518)
06#	0.135	0.151	0.032050	(0.012814, 0.288615)	(−0.051273, 0.352702)
07#	0.106	0.098	0.033753	(−0.047085, 0.243370)	(−0.108916, 0.305202)
08#	0.054	0.040	0.032050	(−0.097996, 0.177805)	(−0.162083, 0.241892)
09#	0.071	0.069	0.032050	(−0.068519, 0.207281)	(−0.132607, 0.271369)

## Data Availability

The original contributions presented in this study are included in the article. Further inquiries can be directed to the corresponding author.
